# Necessity of Antibiotics following Simple Exodontia

**DOI:** 10.1155/2016/2932697

**Published:** 2016-03-27

**Authors:** Waqas Yousuf, Moiz Khan, Hasan Mehdi, Sana Mateen

**Affiliations:** ^1^Department of Oral Surgery, Fatima Jinnah Dental Hospital, Building No. 1, 100 Foot Road, Azam Basti, Karachi 75500, Pakistan; ^2^Department of Oral Pathology, Fatima Jinnah Dental Hospital, Building No. 1, 100 Foot Road, Azam Basti, Karachi 75500, Pakistan; ^3^Fatima Jinnah Dental Hospital, Building No. 1, 100 Foot Road, Azam Basti, Karachi 75500, Pakistan

## Abstract

*Introduction.* The aim of our study is to assess the need for postoperative antibiotics following simple exodontia and determine its role in minimizing patient discomfort and postoperative complications.* Material and Methods.* All the patients undergoing simple extractions were grouped into two categories: Group 1, patients receiving antibiotics, and Group 2, patients receiving no antibiotics. Patients were recalled on the sixth day to assess postoperative complications. On recall, patients were evaluated for signs of persistent inflammation and signs of dry socket. Presence of persistent inflammation and/or suppuration on the 6th day was considered as wound infection.* Results.* A total of 146 patients were included in this study. Out of the total sample, 134 (91.8%) presented with no postoperative complications and 12 (8.2%) had postoperative complications, out of which 11 (7.5%) patients presented with dry socket (alveolar osteitis), 5 (3.4%) in the antibiotic group and 6 (4.1%) in the nonantibiotic group. Only 1 patient (0.7%) was reported with infection of the extraction socket in the nonantibiotic group, whereas no case of infection was found in the antibiotic group.* Conclusion.* Antibiotics are not required after simple extractions in patients who are not medically comprised nor do they have any role in preventing postoperative complications.

## 1. Introduction

The oral cavity has one of the most diverse spectrums of bacterial flora in the body [1, 2]. When left unchecked, it can contribute to local and systemic ill-health [3]. The potential for development of devastating infections has made antibiotics one of the most commonly prescribed drugs in dentistry. Their use is justified in certain cases such as severe pericoronitis, cellulitis, facial space infections, and osteomyelitis [4], whereas other routine dental situations, such as periapical abscess, mild pericoronitis, dry socket, and restorative dentistry [4], do not usually justify the use of antibiotics.

A new class of antibiotics has not been discovered since the 1980s. Indiscriminate use of the current generation of antibiotics has led to the propagation of various resistant organisms [5]. It is therefore imperative that the use of antibiotics be strictly preserved for use only where specifically indicated. Dental prescriptions may account for as much as 7–9% of total antibacterial prescriptions in primary care in some settings [6]. This places a heavy burden of responsibility on dental surgeons to use antibiotics very selectively where indicated and not simply as a routine prophylaxis.

Prescription of antibiotics after simple tooth extraction has remained a controversial topic amongst dental academia. Antibiotics are thought to increase postoperative comfort following exodontia by preventing wound infection and therefore pain. Although bacteremia certainly occurs during simple exodontia [7], it also occurs during many other routine dental procedures in which there is no justification for antibiotic therapy. This is because the body's host response is more than sufficient to counter this level of bacteremia.

The current trend in dentistry in the developed world is shifting to the notion that antibiotics are not justified following simple exodontia [8]. However, surprisingly, little work has been done on this topic in the developing world where standards of oral care are far below those of the developed world. The value of antibiotic therapy in this part of the world has been questionable as the general consensus amongst dental surgeons is that antibiotics are essential to minimize postoperative complications. This trend is exacerbated by patients demand for and often self-prescription of antibiotics even in circumstances where antibiotic therapy is clearly not indicated.

The aim of our study is to assess the need for postoperative antibiotics following simple exodontia and determining its role in minimizing patient discomfort and postoperative complications.

## 2. Material and Methods

*Design.* The design is randomized control trial.

*Setting.* The setting is Fatima Dental Hospital.

*Sample Size.* The sample size is 146 {*n* = 4*pq*/*L* ^2^}.

*Sampling Method.* The sampling method used is nonprobability purposive sampling.

*Purposive Sampling.* All patients undergoing simple tooth extractions from April 2015 till August 2015 were recruited in the study.

*Inclusion Criteria.* Inclusion criteria are as follows: (1) both male and female patients; (2) patients aged between 10 and 80 years; (3) patients with a good systemic health; (4) patients undergoing simple extractions; (5) patients undergoing extractions of permanent mandibular and/or maxillary teeth; (6) patients undergoing single extractions; (7) extractions requiring minimal instrumentation.

*Exclusion Criteria.* Exclusion criteria are as follows: (1) patients undergoing surgical extractions; (2) patients with deciduous teeth; (3) patients with impacted mandibular third molars; (4) patients with a debilitating systemic disease; (5) patients undergoing extractions of endodontically treated teeth; (6) patients currently taking antibiotics at the time of extraction or have had antibiotics less than 3 days prior to extraction; (7) patients with habits which are known to be detrimental to oral health such as smoking, pan, chalia, and/or tobacco chewing; (8) patients presenting with an acute abscess; (9) pregnant patients.

*Data Collection.* All patients who fulfilled the inclusion criteria, after approval by the Institutional Ethical Review Committee were included in this study.

All extractions were performed in the oral surgery department at Fatima Jinnah Dental Hospital by senior dental surgeons (residents) using the following surgical protocol: regular surgical gloves and masks were worn for every extraction; polythene sheets were used to cover each surgical unit and sodium hypochlorite (5%) was used as a potent disinfectant to clean each unit between patients; no more than 2 cartridges of 1.8 mL 2% lidocaine containing 1 : 100,000 epinephrine were administered using 25/27 gauge needle prior to extraction; inferior alveolar nerve block was used for mandibular molars and premolars and local infiltration was used for mandibular anterior teeth and all maxillary teeth.

Extractions were performed with minimal instrumentation using a mucoperiosteal elevator, straight elevator (when required), and forceps. Hemostasis was achieved using a cotton pressure pack. Postoperative instructions were given to every patient, in which the patients were asked to apply pressure on the cotton pack for at least half an hour, were asked to refrain from spitting, rinsing, and sucking, and were also advised to take a soft diet and avoid hot food for at least 24 hours following the extraction.

Patients were recalled after five days to assess postoperative complications including inflammation, wound infection, and dry socket. Evaluation of pain was done using a numeric scale in association with illustrations using charts. These charts were given to patients for self-assessment after every 1-, 6-, 12-, 24-, 48-, and 72-hour intervals in which they were asked to rate the degree of pain. On recall, patients were evaluated for signs of persistent inflammation (i.e., level of pain, swelling, and redness) and signs of dry socket (i.e., presence of denuded bone at the base of the socket accompanied with severe pain). Presence of persistent inflammation and/or suppuration on the 6th day was considered as wound infection.

All the patients undergoing simple extractions were grouped into two major categories:

 Group 1: patients receiving antibiotics.
 Group 2: patients receiving no antibiotics.

### 2.1. Group 1: Patients Receiving Antibiotics

All patients in this group were prescribed amoxicillin with clavulanic acid 625 mg 12 hourly for 5 days along with flurbiprofen 100 mg 8 hourly for 3 days starting 30 minutes after the extraction.

### 2.2. Group 2: Patients Not Receiving Antibiotics

All patients in this group were not prescribed antibiotic and were given flurbiprofen 100 mg 8 hourly for 3 days starting 30 minutes after the extraction.

Randomization was achieved using the closed envelope technique. In this randomization technique, dental surgeons were given randomly generated prescription regimen within sealed opaque envelopes. After establishing consent, the envelope was opened and the patient was then offered the allocated prescription regimen.

*Data Analysis.* Data was analyzed using SPSS version 21. Chi square test was used to test the *p* value.

*Null Hypothesis.* Antibiotics do not significantly reduce postoperative complications in young healthy patients following simple tooth extraction.

## 3. Results

**Figure 1:**
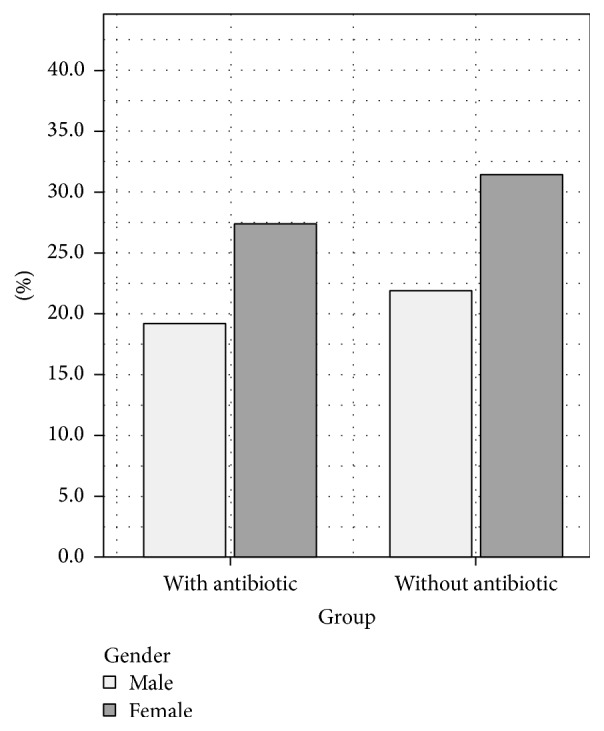
Gender distribution.

Out of the initial sample of 250 (125 in each group), 146 patients appeared for follow-up appointment, out of which 60 (41.1%) were males and 86 (58.9%) were females. Antibiotic group comprised 68 patients (28 males and 40 females) and nonantibiotic group included 78 patients (32 males and 46 females). See Figure 1.

**Figure 2:**
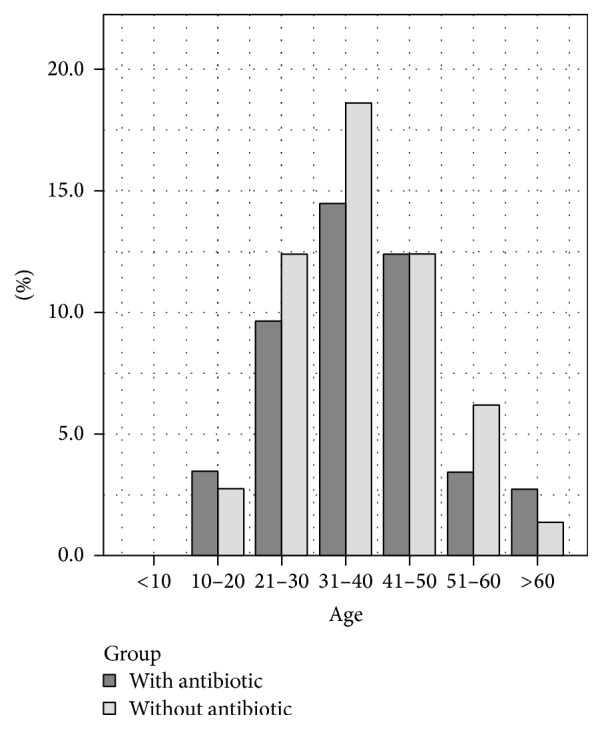
Age distribution.

Out of the total sample, 65 were maxillary teeth and 81 mandibular teeth. The mean age of the patients was 38.46 ± 12.81. In males the mean age was 40.88 ± 13.87 and in females the mean age was 36.75 ± 11.79. See Figure 2.

**Figure 3:**
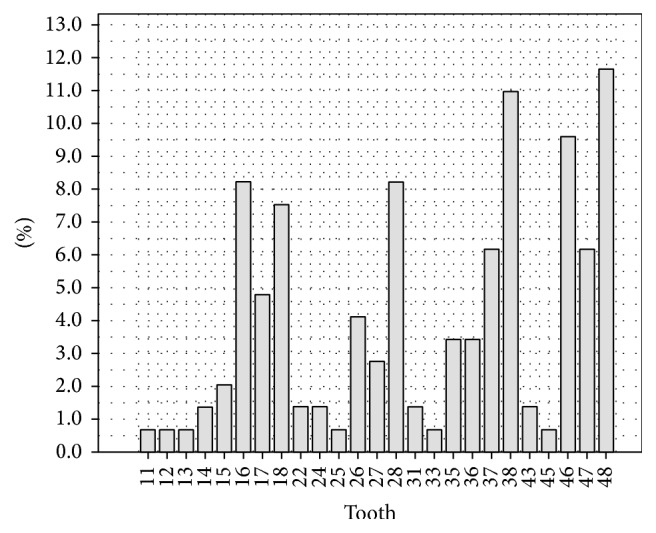
Extracted teeth.

The most commonly extracted teeth were mandibular third molars 22.6% (11.6% right mandibular third molars and 11.0% left mandibular third molars) followed by maxillary third molars 15.7% (7.5% right maxillary third molars and 8.2% left maxillary third molars). See Figure 3.

**Figure 4:**
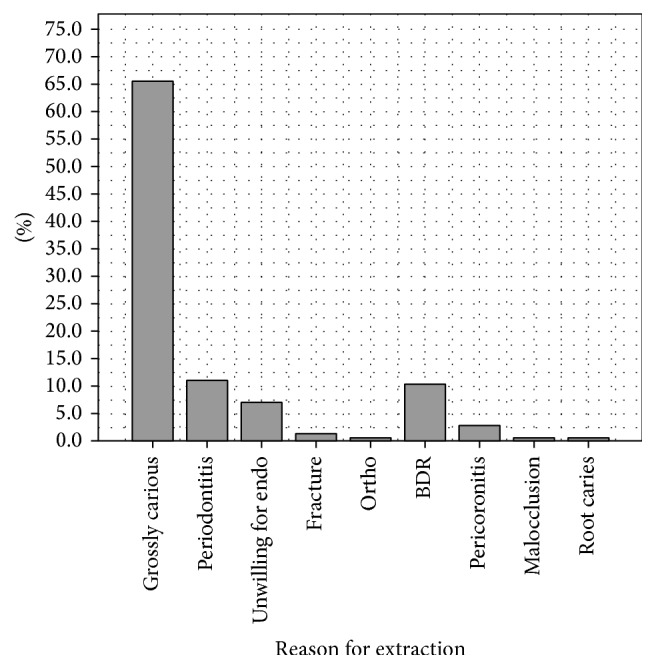
Reasons for extractions.

In the total sample, the most common reason for extraction was that they were found to be grossly carious 65.5%, followed by periodontitis 11.0% and broken down roots 10.3%. See Figure 4.

The mean time taken for extraction was 14.51 minutes ± 9.98.

Out of the total sample, 134 (91.8%) presented with no postoperative complications and 12 (8.2%) had postoperative complications, out of which 11 (7.5%) patients presented with dry socket (alveolar osteitis), 5 (3.4%) in the antibiotic group and 6 (4.1%) in the nonantibiotic group. Only 1 patient (0.7%) was reported with infection of the extraction socket in nonantibiotic group, whereas no case of infection was found in the antibiotic group.

Out of the 11 cases of dry socket, interestingly, 10 (90.9%) cases belonged to females, whereas only 1 (9.1%) was found in males. Although there was no relationship between antibiotic use and dry socket (see Table 1), the overall female predisposition was found statistically significant (*p* = 0.025).

Out of the total sample, 10 (6.9%) patients showed at least one adverse effect to the drugs prescribed. Diarrhea was reported by 5 (3.4%) patients, abdominal discomfort by 3 (2.1%) patients, and vomiting by 2 (1.4%) patients. The vast majority of patients, 9 (90.0%), who presented with an adverse effect belonged to the antibiotic group. Only 1 patient from the nonantibiotic group reported an adverse effect (vomiting). See Table 2. The relationship between adverse effects and antibiotics was proved to be statistically significant (*p* = 0.021).

Out of all the patients who experienced an adverse effect, only 2 out of 10 were males. However, this female predisposition was found to be statistically insignificant (*p* = 0.471).

Out of the total sample, 142 patients correctly filled out the pain chart. The overall trend showed the decrease in preoperative pain after the first hour followed by a slight increase after 6 hours and then a gradual decline over the next 5 days. This trend was equally represented in both groups although to varying degrees. See Tables 1–3. A one-way repeated analysis of variance (ANOVA) determined that the mean pain score has been statistically significant between assessment stages (6 hrs, 12 hrs, 24 hrs, 48 hrs, and 72 hrs) showing a greater drop in the antibiotic group over 72 hours.

Out of the total sample, 103 (45 in the nonantibiotic and 54 in the antibiotic group) patients presented with a complaint of preoperative pain ranging from very mild to very severe pain. Out of 103 patients, 24 (13 in the nonantibiotic and 11 in the antibiotic group) patients reported postoperative pain ranging from very mild to very severe even after 6 days. The average preoperative pain was 5.26 ± 3.66 in the antibiotic group and 3.64 ± 3.49 in the nonantibiotic group.

Out of the 11 patients who presented with postoperative dry socket, 10 (90.9%) were patients with dry socket who reported preoperative pain (11.2% of all cases with preoperative pain) (see Table 4). Only one case of dry socket occurred in patients who reported no preoperative pain (2.4% of all cases who presented without any preoperative pain). However, this relationship was found to be statistically insignificant (*p* = 0.111). See Table 3.

Interestingly, the mean preoperative pain amongst patients reported to be hypertensive was 6.31 compared with an overall average of 4.42. However, this finding was statistically insignificant (*p* = 0.515).

## 4. Discussion

The results unequivocally point towards the use of antibiotics following extractions as meaningless. This was proved by the fact that there was only a solitary case of infection amongst the entire sample. These findings are in agreement with numerous other studies such as those done by van Eeden and Bütow [9] and Agrawal et al. [10]. Conversely, these findings were in contrast to a study done by Arteagoitia et al. [11], who reported a significant rise in the rate of infection related complications in individuals who were not prescribed antibiotics (up to 12.9%). However, it should be mentioned that the aforementioned study was done exclusively on impacted molars and therefore may have limited bearing on the present study. That is not to say that there were no postoperative complications in the present study. A number of patients presented with dry socket and postoperative pain even upon evaluation on the 6th day (see Table 1). Predictably, the number of diagnosed dry socket cases was almost evenly distributed in both groups. This is not surprising as dry socket is a phenomenon which relates to lack of clot retention/formation within the socket and is not considered an infectious process. These findings correlate with other studies conducted by Arteagoitia et al. [11] and López-Cedrún et al. [12] which noted no difference in prevalence of dry socket when postoperative antibiotics were given. However, it should be noted that in a study conducted by van Eeden and Bütow [9], there were no cases of dry socket in individuals who were given antibiotics, whereas 15.8% of those who were not given antibiotics presented with dry socket.

Interestingly, the vast majority of dry socket cases were reported in females. Conversely, males showed a comparatively negligible incidence of dry socket. This statistically significant female predisposition is surprising and difficult to explain. Dry socket is caused by many factors such as traumatic extractions [13] and dislodgment or inability of a clot to from. These factors tend to be evenly distributed in both genders, especially considering that none of the females had a personal or family history of bleeding disorder nor were they taking any substances (such as oral contraceptives or anticoagulants) which could affect the clotting process. Therefore, lacking any systemic cause, this female predisposition seems to be linked with local causes of clot dislodgement and hence can perhaps be attributed to postoperative complication not being followed attentively by females. In another study, two-thirds of the cases of dry socket belonged to males [9], further mystifying the cause of a significant female predisposition in the present study.

Although all drugs are known to have adverse effects, unsurprisingly, patients belonging to antibiotics group reported more adverse effects when compared with their counterparts in the nonantibiotic group (see Table 2). These effects were predominantly related to gastrointestinal tract and included diarrhea, abdominal pain, and vomiting. Although these cases presented in only a small minority of patients who consumed antibiotics, they still bring into question the use of antibiotics unnecessarily without producing any tangible benefits. In fact this increases the physical as well as financial burden on the patient. This is especially a problem in developing countries where it is difficult for patients to afford an antibiotic regimen in addition to treatment. In a country like Pakistan where the daily wage is below the poverty line, adverse effect of antibiotics can result in lost wages and can significantly disrupt their daily life. On a community level the overuse of antibiotic has many consequences such as promoting the development of resistant organisms [14]. They may also be associated with unfavorable drug interactions as demonstrated by Hersh [15]. Dentists have an ethical responsibility to play their role in preventing the propagation of such microbes by limiting the use of antibiotics and being selective in their prescription.

**Figure 5:**
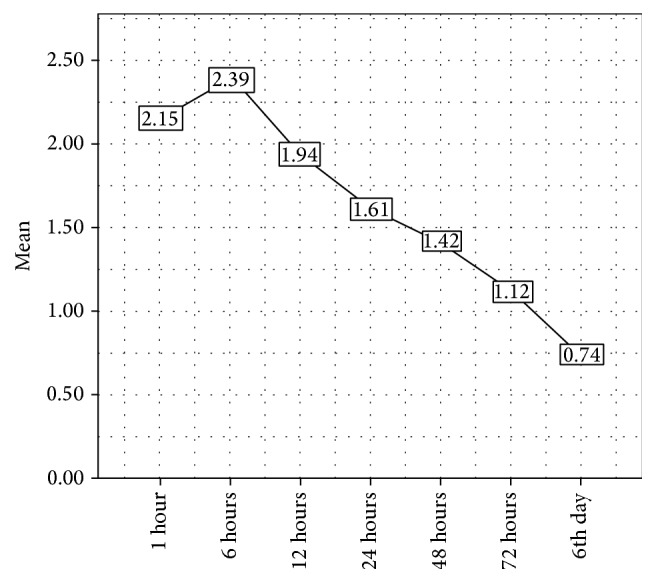
Overall pain trend over 6 days in both groups.

**Figure 6:**
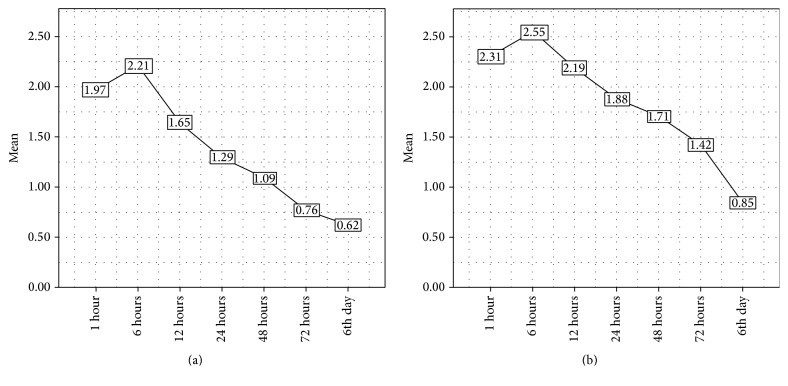
Pain trend over 6 days in the antibiotic (a) and nonantibiotic (b) groups.

The antibiotic group showed a better pain profile than the nonantibiotic group, showing a steeper decline in pain despite having a higher mean preoperative pain (see Figures 5 and 6). This finding is in contrast with studies conducted by van Eeden and Bütow [9] and Agrawal et al. [10], who reported no significant relationship between the use of antibiotics and postoperative pain. However, it should be noted that despite being statistically significant (*p* = 0.002) in the present study, this effect was clinically trifling and therefore does not justify the use of antibiotics. The vast majority of dental practitioners in this region routinely prescribe antibiotics as a preventive measure to avoid postoperative complications, namely, pain and infection. This practice must be stopped as the evidence overwhelmingly proves that this is unacceptable and a disservice to not only the patient but also the community at large. The use of a stronger analgesic is a much better option after simple extractions to reduce pain in lieu of antibiotics [16].

## 5. Conclusion

Antibiotics are not required after simple extractions in patients who are not medically comprised nor do they have any role in preventing postoperative complications. Dental practitioners must show greater responsibility and selectivity when prescribing antibiotics.

## Figures and Tables

**Table 1:** Distribution of dry socket amongst males and females in both groups.

	Dry socket	
Male		
Group I	27 (1)—3.7%	*χ* ^2^ 1.17, *p* value = 0.467
Group II	32 (0)—0.0%	
Female		
Group I	36 (4)—11.1%	*χ* ^2^ 0.193, *p* value = 0.745
Group II	40 (6)—15.0%	

Group I: with antibiotics.

Group II: without antibiotics.

**Table 2:** Drug adverse effects in both groups.

Adverse effects	With antibiotic	Without antibiotic
None	59	77
Diarrhea	5	0
Stomach pain	3	0
Vomiting	1	1

**Table 3:** Mean distribution of postoperative pain in both groups.

Study groups	Assessment stages	Mean	SD	*n*
With antibiotics	6 h	2.21	1.897	68
	12 h	1.65	1.726	68
	24 h	1.29	1.497	68
	48 h	1.09	1.743	68
	72 h	0.729	1.729	68

Without antibiotics	6 h	2.55	2.66	78
	12 h	2.19	2.53	78
	24 h	1.88	2.39	78
	48 h	1.71	2.53	78
	72 h	1.42	2.63	78

**Table 4:** Association of preoperative pain with dry socket.

Preoperative pain	Dry socket	
	Yes	No
Yes	10	89
No	1	42

## References

[1] Samarnayake L. (2006). *Essential Microbiology for Dentistry*.

[2] Parahitiyawa N. B., Scully C., Leung W. K., Yam W. C., Jin L. J., Samaranayake L. P. (2010). Exploring the oral bacterial flora: current status and future directions. *Oral Diseases*.

[3] Seymour G. J., Ford P. J., Cullinan M. P., Leishman S., Yamazaki K. (2007). Relationship between periodontal infections and systemic disease. *Clinical Microbiology and Infection*.

[4] Hupp J. R., Elis E., Tucker M. R. (2008). *Contemporary Oral and Maxillofacial Surgery*.

[5] Levy S. B. (2001). Antibiotic resistance: consequences of inaction. *Clinical Infectious Diseases*.

[6] Karki A. J., Holyfield G., Thomas D. (2011). Dental prescribing in Wales and associated public health issues. *British Dental Journal*.

[7] Lockhart P. B., Brennan M. T., Sasser H. C., Fox P. C., Paster B. J., Bahrani-Mougeot F. K. (2008). Bacteremia associated with toothbrushing and dental extraction. *Circulation*.

[8] Bortoluzzi M. C., Manfro R., De Déa B. E., Dutra T. C. (2010). Incidence of dry socket, alveolar infection, and postoperative pain following the extraction of erupted teeth. *The Journal of Contemporary Dental Practice*.

[9] van Eeden S. P., Bütow K. (2006). Post-operative sequelae of lower third molar removal: a literature review and pilot study on the effect of Covomycin D. *SADJ*.

[10] Agrawal M., Rahman Q. B., Akhter M. (2012). Extraction of asymptomatic tooth with and without antibiotic therapy. *Bangabandhu Sheikh Mujib Medical University Journal*.

[11] Arteagoitia I., Diez A., Barbier L., Santamaría G., Santamaría J. (2005). Efficacy of amoxicillin/clavulanic acid in preventing infectious and inflammatory complications following impacted mandibular third molar extraction. *Oral Surgery, Oral Medicine, Oral Pathology, Oral Radiology and Endodontology*.

[12] López-Cedrún J. L., Pijoan J. I., Fernández S., Santamaria J., Hernandez G. (2011). Efficacy of amoxicillin treatment in preventing postoperative complications in patients undergoing third molar surgery: a prospective, randomized, double-blind controlled study. *Journal of Oral and Maxillofacial Surgery*.

[13] Oginni F. O. (2008). Dry socket: a prospective study of prevalent risk factors in a Nigerian population. *Journal of Oral and Maxillofacial Surgery*.

[14] Yoshikawa T. T. (2002). Antimicrobial resistance and aging: beginning of the end of the antibiotic era?. *Journal of the American Geriatrics Society*.

[15] Hersh E. V. (1999). Adverse drug interactions in dental practice: interactions involving antibiotics. *Journal of the American Dental Association*.

[16] Al-Khateeb T. H., Alnahar A. (2008). Pain experience after simple tooth extraction. *Journal of Oral and Maxillofacial Surgery*.

